# Guanidinoacetic acid regulated postmortem muscle glycolysis associated with AMPK signaling and protein acetylation

**DOI:** 10.5713/ab.24.0418

**Published:** 2024-10-28

**Authors:** Ning Liu, Bolin Zhang, Shubai Wang, Qingzhen Zhong, Zewei Sun

**Affiliations:** 1 College of Animal Science and Technology, Jilin Agricultural University, Changchun 130118, China; 2 College of Animal Science and Technology, Qingdao Agricultural University, Qingdao 266109, China; 3 Department of Biology and Agriculture, Zunyi Normal College, Zunyi 563006, China

**Keywords:** Adenosine 5'-Monophosphate-Activated Protein Kinase (AMPK) Signaling, Antemortem Stress, Glycolysis, Guanidinoacetic Acid, Protein Acetylation

## Abstract

**Objective:**

Antemortem stress accelerated muscle energy consumption in postmortem muscle. The objective of our study was to investigate the regulation of guanidinoacetic acid (GAA) administration on the postmortem glycolysis and protein acetylation in postmortem muscle of antemortem stress.

**Methods:**

Forty C57BL/6 male mice were chosen and randomly assigned to four treatment groups (A, B, C and D), each treatment consisted of 10 replicates. Mice in group B, C and D were treated with 0.05% GAA oral administration for 6 days. On the 7th day of the experiment, the mice in group A and B were injected with saline, and mice in group C and D were injected with 5-aminoimidazole-4-carboxamide1-β-D-ribofuranoside (AICAR; 50 μg/g body weight) and a combined injection with AICAR (50 μg/g body weight) and histone acetylase inhibitor II (HAT II; 185 μg/g body weight), respectively.

**Results:**

The results showed that the values of pH_45min_ and pH_24h_ of postmortem muscle in GAA administration were higher than those in the control group. However, the opposite result was observed in AICAR group. Moreover, the activities of pyruvate kinase, hexokinase and fruc-tose-2,6-diphosphatase, combined with the protein abundance of phosphorylated liver kinase, phosphorylated AMPKα2 and total acetylated protein were all decreased by GAA administration and HAT II treatment.

**Conclusion:**

Taken together, adenosine 5'-monophosphate-activated protein kinase signaling and protein acetylation could mediate the regulation of GAA administration on postmortem glycolysis of antemortem stress-muscle.

## INTRODUCTION

Adenosine 5'-monophosphate-activated protein kinase (AMPK), composed of three different subunits (α, β, and γ), is considered as a key regulator of energy metabolism [1] and is activated in response to the increased ratio of adenosine monophosphate (AMP) to adenosine triphosphate (ATP) [2]. Previous studies indicated that AMPK plays a crucial role in glycolysis of postmortem skeletal muscle [3,4]. After slaughter, the circulation oxygen supply was reduced, followed by the main metabolism of skeletal muscle from aerobic to anaerobic. In this case, the glycogen stored in postmortem muscle was transferred into lactic acid (LD), resulting in an accumulation of LD in muscle and a sharp decline in the final pH of meat at postmortem 24 h [5]. Additionally, accumulated evidences revealed that pre-slaughter stress is one of the two main factors leading to the occurrence of pale, soft, exudative (PSE) meat [6,7]. Pre-slaughter stress accelerated glycolysis metabolism and upregulated the activities of hexokinase (HK), pyruvate kinase (PK) and lactate dehydrogenase (LDH), which are involved in glycolysis of postmortem muscle [5,8]. Moreover, it was reported that pre-slaughter handling might be associated with glycolysis in postmortem muscle mediated by protein acetylation [9].

Guanidinoacetic acid (GAA), acting as a natural precursor of creatine (Cr), can generate ATP through the Cr/phosphocreatine (PCr) system [10]. GAA is mainly synthesized in the kidney and transported into the liver to yield Cr, followed by the release of Cr into the circulation and the transport of Cr into the target tissue [11]. Moreover, it has been demonstrated that GAA can be more effective than Cr at increasing Cr stores in tissues [12,13]. GAA administration can also amplify the availability of Cr in skeletal muscle through enhancing Cr biosynthesis [13]. In addition, it was reported that the concentrations of GAA and Cr in serum were enhanced after a six-week oral GAA administration [14]. However, previous studies in broilers suggested that the concentrations of Cr and PCr dramatically decreased due to acute and chronic physiological stimuli, such as heat stress and transport stress [15,16]. It has been documented that GAA administration contributed to enhancing the Cr/PCr content in muscle [12,13] and attenuating the pre-slaughter transport-induced muscle energy expenditure and rapid glycolysis of muscle [17]. Therefore, under the condition of stress, it may be an effective measure to elevate muscle energy stores in order to compensate for stress-induced high energy consumption and alleviate the glycolysis of postmortem muscle to decrease the occurrence of PSE meat. However, to the best of our knowledge, the regulation of GAA on energy metabolism of postmortem muscle subjected to pre-slaughter stress is still unclear. Therefore, we hypothesized that GAA could regulate postmortem glycolysis of antemortem-stress muscle associated with AMPK signaling and protein acetylation.

In the present study, a model using mice was conducted with GAA administration combined with/without some activator or acetyltransferase was used to investigate the effect of GAA administration on postmortem energy status and the glycolysis in antemortem-stressed muscle, and further reveal the mechanism of GAA attenuating postmortem meat glycolysis through AMPK signaling pathway and protein acetylation, which could provide theoretical basis for nutrition regulation of energy metabolism in postmortem muscle of livestock animals subjected to pre-slaughter stress and contribute to providing guarantees for high-quality meat production.

## MATERIAL AND METHODS

### Experimental design

All the procedures in the present study were approved by the Institutional Animal Care and Use Committee of Zunyi Normal College (Zunshi[2022]06).

**Figure 1:**
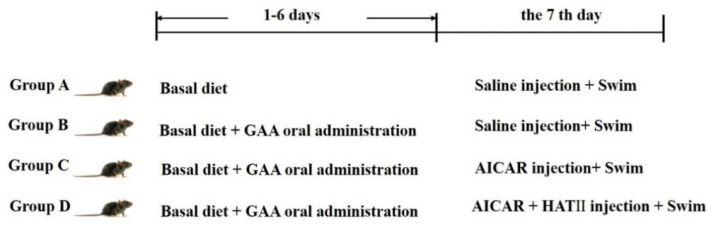
The flowchart of this study. During 1–6 days of the experiment, all rats were fed with the basal diet. Rats in group B, C and D were also orally administrated with GAA at a dose of 0.05 g/kg body weight. At the 7th day of the experiment, rats in group A and B were injected with saline, however, rats in group C and D were separately treated with AICAR (50 μg/g body weight) and a combination injection with AICAR (50 μg/g body weight) and HAT II (185 μg/g body weight). GAA, guanidinoacetic acid; AICAR, 5-aminoimidazole-4-carboxamide1-β-D-ribofuranoside, HAT II, histone acetylase inhibitors II.

A total of forty C57BL/6 male mice (3 months old) with similar weights were obtained and randomly assigned to four treatment groups (A, B, C and D). Each treatment consisted of ten replicates. Mice in group A were fed with the basal diet, while groups B, C and D were fed with the basal diet and treated with GAA oral administration (0.05 g/kg body weight) for six days. The source of the GAA additive (> 99% purity) was from Tianjin Tiancheng Pharmaceutical Co., Ltd. (Tianjin, China) and the dose of GAA administration was chosen according to Ostojec et al [18]. All mice were housed in an environmentally controlled facility and were given *ad libitum* access to feed and fresh water. At the end of the experiment, all mice were treated according to the following procedures: mice in group A (defined as the control group) and group B were injected with 0.9% saline solution, however, mice in group C in group D were intraperitoneally injected with 50 μg/g body weight 5-aminoimidazole-4-carboxamide1-β-D-ribofuranoside (AICAR, a specific activator of AMPK) and intraperitoneally injected with a combine of 50 μg/g body weight AICAR and 185 μg/g body weight histone acetylase inhibitors II (HAT II) [7,19] . The doses of AICAR and HAT II used in our study were according to a previous study [7]. After waiting for 15 min following the injection, all mice were forced to swim for 2 min to simulate antemortem-stress [19,20]. All mice were anaesthetized by CO_2_ and killed by cervical dislocation. The design of the specific treatment groups is shown in Figure 1.

### pH measurement

About 0.20 g longissimus dorsi muscle was taken within 5 min after slaughter and stored at 4°C for the determination of postmortem pH. The measurement of pH was conducted with a portable digital pH meter (PHBF-260; Shanghai Instrument Electric Science Instrument Co., Ltd., Shanghai, China). The ultimate pH value of postmortem longissimus dorsi muscle refers to the pH value at 24 h postmortem [1,21].

### The activities of hexokinase, pyruvate kinase, and fructose-2,6-diphosphatase

The enzyme activities of HK, PK and PFK were all conducted with commercial kits (Nanjing Jiancheng Bioengineering Institute, Nanjing, China). All operation protocols were according to instructions of manufactures.

### The contents of adenosine phosphates

The concentrations of ATP, adenosine diphosphate (ADP) and AMP in muscle were determined by HPLC according to our previous report with moderate modifications [15]. Eighty mg frozen muscle collected into a tube with ice-cold perchloric acid were homogenized for 1 min, followed by a 15 min standing in an ice bath. Afterwards, the homogenates were centrifuged at 15,000×g at 4°C for 10 min to collect supernatants and further filtered through a 0.45-μm membrane. The extracted sample was separated by Alliance HPLC system (Alliance HPLC system 2695; Waters Corporation, Milford, MA, USA) equipped with a Waters SunFire C18 column (250 mm×4.6 mm, 5 μm) for ATP, ADP and AMP analysis at 245 nm. The standards of 5-ATP disodium salt, 5-ADP sodium salt, and 5-AMP sodium salt were all purchased from Sigma-Aldrich, Inc. (St. Louis, MO, USA).

### Western-blot analysis

Fifty mg frozen muscle samples were homogenized in tubes containing 200 μL lysis buffer and centrifuged for 10 min at 6200 ×g, 4°C to collect the supernatant. Appropriate 40 μg protein was separated by 12% sodium dodecyl sulfate polyacrylamide gel and subsequently transferred to a polyvinylidene difluoride membrane (Millipore, Billerica, MA, USA), followed by a block handling with 5% bovine serum albumin for 1 h and an incubation with primary antibodies (1:1,000) overnight at 4°C. After three times washes with (1×Tris buffered saline including 0.1% Tween 20), the membranes were incubated with horseradish peroxidase-conjugated second antibody (1:3,000; Cell Signaling Technology Inc., Beverly, MA, USA) at room temperature. The primary antibodies were phosphor liver kinase B1 (LKB1, Thr189, no. 3054s), phosphor-AMKPα (Thr172, no. 2531s), rabbit acetylated-lysine antibody (no. 9441) and glyceraldehyde-3-phosphate dehydrogenase (GAPDH; no. 97166), and all primary antibody were purchased from Cell Signaling Technology Inc. and diluted at 1:1,000. The membranes were developed with ECL chemiluminescence reagents (Tanon Science and Technology Co., Ltd., Shanghai, China) and visualized with a Tanon 4600 fully automatic chemiluminescence image analysis system (Tanon Science and Technology Co., Ltd.). The expressions of the target proteins mentioned above were normalized to GAPDH and expressed as the values relative to those for the control group.

### Statistical analysis

All data were statistically analyzed with the SPSS statistical software (version 20.0). All data were normally distributed and analyzed using one-way analysis of variance. Significant differences among different treatments were further analyzed by Duncan’s multiple-range test. The results were presented with mean values and their standard deviation, and p<0.05 was considered statistically significant.

## RESULTS

### Effect of oral administration with guanidinoacetic acid on pH value

**Figure 2:**
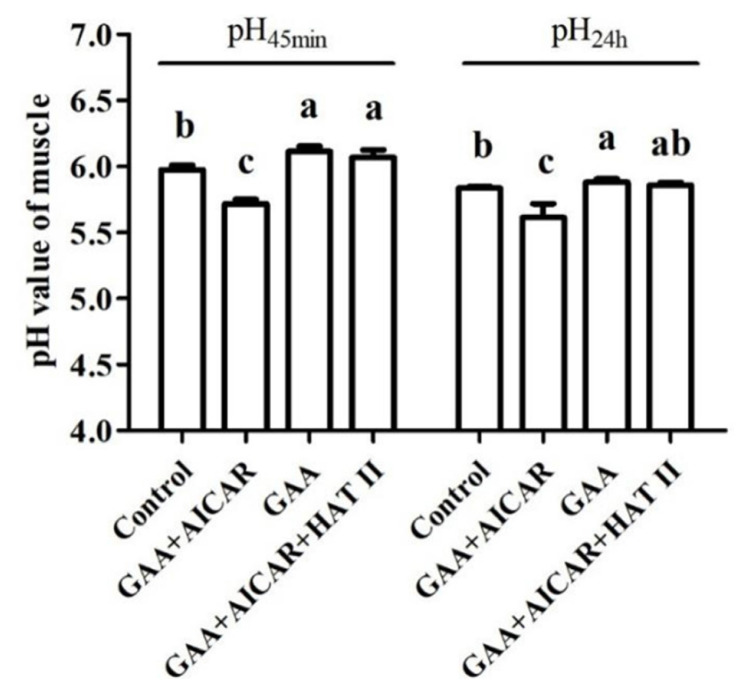
Effect of guanidinoacetic acid administration on pH of postmortem muscle. Control, mice were fed the basal diet; GAA+AICAR, mice were fed the basal diet and treated with GAA oral administration combined with intramuscular injection with AICAR; GAA, mice were fed the basal diet and treated with GAA oral administra-tion; GAA+AICAR+HAT II, mice were fed the basal diet and treated with GAA oral administra-tion combined with intramuscular injection with AICAR and HAT II. pH_45min_, pH value at post-mortem 45 min; pH_24h_, pH value at postmortem 24 h; GAA, guandine acetic acid; AICAR, 5-aminoimidazole-4-carboxamide1-β-D-ribofuranoside; HAT II, histoneacetyltransferase II. The given values are expressed as mean value±standard deviation (n = 10). ^a–c^ Bars with different small letters mean significant difference (p<0.05).

As shown in Figure 2, compared with the control group, oral administration with GAA significantly increased the values of pH_45min_ and pH_24h_ of the postmortem skeletal muscle (p<0.05). In contrast, AICAR injection lowered the values of pH_45min_ and pH_24h_ (by 4.51% decrease) of the postmortem skeletal muscle in comparison with those treated with GAA oral administration (p<0.05). However, compared with those that received an AICAR injection, mice receiving both AICAR and HAT II treatment exhibited a higher value of pH_45min_ and pH_24h_ (by 4.32% increase) of the postmortem skeletal muscle (p<0.05). No differences were observed between the GAA group and the AICAR and HAT II combined treatment group (p>0.05). In addition, compared with those in the control group, the pH_45min_ value of postmortem muscle was significantly increased by AICAR and HAT II injection (p<0.05), but the pH_24h_ value was not affected (p>0.05).

### The concentration of adenosine phosphates in muscle

As displayed in Table 1, GAA oral administration increased the ATP content (by 6.37% increase), but lowered the contents of ADP and AMP and the ratio of AMP to ATP (p<0.05). The lowered ATP content (by 4.93% decrease) and the increased contents of ADP and AMP, combined with the higher ratio of AMP to ATP were observed in the AICAR group compared with the GAA group (p<0.05). In contrast, AICAR+HAT II injection decreased the content of AMP and the ratio of AMP to ATP compared with those treated with AICAR injection (p<0.05), but there were no differences in the contents of ATP and ADP (p>0.05). In comparison with the GAA group, AICAR+HAT II injection did not affect the contents of ATP, ADP and AMP and the ratio of AMP to ATP (p>0.05).

### The activities of key enzymes in glycolysis

**Figure 3:**
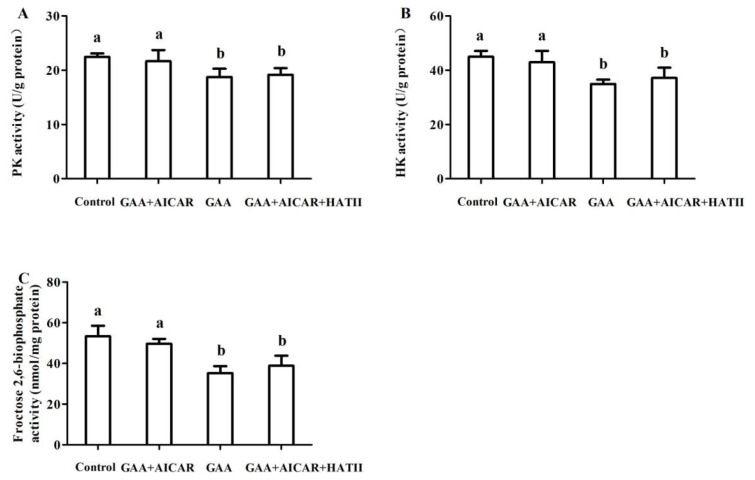
Effect of guanidinoacetic acid administration on the activities of PK (A), HK (B) and Froc-tose-2,6-biophosphate (C) involved in glycolysis of postmortem muscle. Control, mice were fed the basal diet; GAA+AICAR, mice were fed the basal diet and treated with GAA oral administration combined with intramuscular injection with AICAR; GAA, mice were fed the basal diet and treated with GAA oral administra-tion; GAA+AICAR+HAT II, mice were fed the basal diet and treated with GAA oral administra-tion combined with intramuscular injection with AICAR and HAT II. PK, pyruvate kinase; GAA, guanidinoacetic acid; AICAR, 5-aminoimidazole-4-carboxamide1-β-D-ribofuranoside; HAT II, histoneacetyltransferase II; HK, hexokinase. The given values are ex-pressed as mean value±standard deviation (n = 10). ^a,b^ Bars with different small letters mean significant difference (p<0.05).

As presented in Figure 3, in comparison with the control group, oral GAA administration lowered the activities of PK (Figure 3A), HK (Figure 3B) and PFK (Figure 3C) (p<0.05). But AICAR injection significantly increased the activities of HK, PK and PFK compared with the GAA group (p<0.05). The activities of HK, PK and PFK of mice muscle in the AICAR+HAT II group were lower than those in the AICAR group (p<0.05). There were no differences in the activities of HK, PK and PFK of mice muscle between the AICAR+HAT II group and the GAA group (p>0.05), however, the activities of HK, PK and PFK in the AICAR+HAT II group were lower than the control group (p<0.05).

### Protein expression of adenosine 5'-monophosphate-activated protein kinase signaling pathway

**Figure 4:**
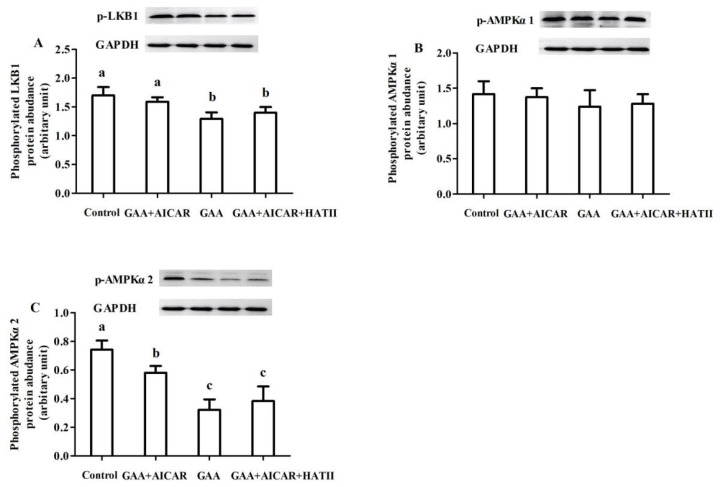
Effect of guanidinoacetic acid administration on protein abundances of LKB1 (A), AMPKα1 (B) and AMPKα2 (C) in AMPK signaling pathway of postmortem muscle. Control, mice were fed the basal diet; GAA+AICAR, mice were fed the basal diet and treated with GAA oral administration combined with intramuscular injection with AICAR; GAA, mice were fed the basal diet and treated with GAA oral administra-tion; GAA+AICAR+HAT II, mice were fed the basal diet and treated with GAA oral administra-tion combined with intramuscular injection with AICAR and HAT II. p-LKB1, phosphorylated liver kinase B1; p-AMPKα1, phosphorylated adenosine 5’-monophosphate--activated protein ki-nase; p-AMPKα2, phosphorylated adenosine 5’-monophosphate--activated protein kinase; GAPDH, glyceraldehyde-3-phosphate dehydrogenase; GAA, guanidinoacetic acid; AICAR, 5-aminoimidazole-4-carboxamide1-β-D-ribofuranoside; HAT II, histoneacetyltransferase II. The given values are ex-pressed as mean value±standard deviation (n = 10). ^a–c^ Bars with different small letters mean significant difference (p<0.05).

The results listed in Figure 4 showed that, in comparison with the control group, oral GAA administration significantly lowered the protein expressions of phosphorylated LKB1 (Figure 4A) and AMPKα2 (Figure 4C) in the postmortem muscle of mice (p<0.05). But the phosphorylation level of AMPKα1 (Figure 4B) was not affected (p>0.05). AICAR injection significantly increased the protein abundance of phosphorylated LKB1 and AMPKα2 compared with the GAA group (p<0.05). On the contrary, compared with those treated with AICAR injection, AICAR+HAT II treatment downregulated the protein expression of phosphorylated LKB1 and AMPKα2 (p<0.05). No differences in the protein expressions of phosphorylated LKB1, AMPKα2 and AMPKα1 of mice muscle were observed between the AICAR+HAT II group and the GAA group (p>0.05). However, the protein expression of phosphorylated LKB1 and AMPKα2 in the AICAR+HAT II group were significantly lower than those in the control group (p<0.05).

### Total protein acetylation level

**Figure 5:**
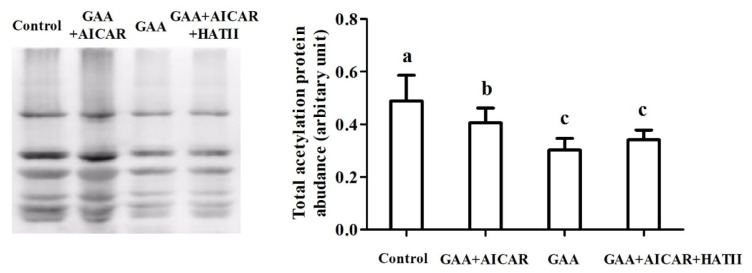
Effect of guanidinoacetic acid administration on the abundances of total acetylated proteins in postmortem muscle. Control, mice were fed the basal diet; GAA+AICAR, mice were fed the basal diet and treated with GAA oral administration combined with intramuscular injection with AICAR; GAA, mice were fed the basal diet and treated with GAA oral administration; GAA+AICAR+HAT II, mice were fed the basal diet and treated with GAA oral administration combined with intramuscular injection with AICAR and HAT II. GAA, guanidino-aceticacid; AICAR,5-aminoimidazole-4-carboxamide1-β-D-ribofuranoside; HAT II, histoneacetyltransferase II. The given values are expressed as mean value±standard deviation (n = 10). ^a–c^ Bars with different small letters mean significant difference (p<0.05).

The results demonstrated that, in comparison with the control group, total protein acetylation protein was lowered in response to GAA oral administration (p<0.05, Figure 5). But AICAR injection significantly elevated total protein acetylation protein expression compared with the GAA group (p<0.05). The total protein acetylation protein expression in AICAR+HAT II group was lower than that in the AICAR group (p<0.05). Additionally, there was no difference in total protein acetylation protein expression of muscle between the AICAR+HAT II group and the GAA group (p>0.05). However, the total protein acetylation protein expression in the AICAR+HAT II group was significantly lower than those in the control group (p<0.05).

## DISCUSSION

Following exsanguination, the oxygen supply via blood circulation was cut off and the tissue metabolism was mainly transferred from an aerobic into an anaerobic condition. The drop of postmortem muscle pH could be ascribed to the accumulation of hydrogen ions (H^+^). The rate of postmortem pH decline is controlled by the rate of ATP hydrolysis catalyzed by muscle ATPs. The depletion of ATP yields H^+^ and results in a progressive fall in pH to the ultimate value of postmortem meat [22]. Nevertheless, it has been demonstrated that Cr monohydrate or GAA administration could enhance the levels of PCr in muscle [2,5], which could transfer a phosphate group from PCr to ADP to generate ATP, thus preventing a depletion of ATP levels [23] and further alleviating the rate of pH decline. In the present study, the values of pH_45min_ and pH_24h_ in the oral GAA administration group were higher than those in the control group. Similar to this, in our previous studies in broilers, GAA supplementation increased the value of pH_24h_ in broiler muscle compared with those subjected to a 3 h pre-slaughter transport stress [2,15]. In comparison with the GAA group, AICAR injection decreased muscle pH value, but no differences were observed between the GAA group and the GAA+AICAR+HAT II group. Similarly, Li et al [7] reported that AICAR injection led to a decrease in muscle pH, however, there were no differences between AICAR+HAT II treatment and the control group. Shen et al [20] also suggested that AICAR treatment induced a significant drop in muscle pH_24h_ in comparison with the control group. These results mentioned above indicate that GAA administration could contribute to alleviating the rate of muscle pH decline.

After slaughter, muscle attempts to maintain cellular ATP levels within homeostatic set points, therefore additional metabolite catabolism is necessary. Pre-slaughter stress accelerated glycolysis metabolism to generate ATP for the increased muscle energy demands in response to the limited oxygen supply in anaerobic glycolysis and then became the predominant energy source for muscle ATP supply [2]. The activities of HK, PK and PFK which are key enzymes involved in glycolysis in postmortem muscle, were decreased in the GAA group, implying that the rate of glycolysis reaction was delayed owing to GAA administration. But compared with the GAA group, AICAR treatment significantly increased the activities of PK, HK and PFK in muscle. It is well-known that AICAR is an activator of AMPK. Accordingly, a previous study suggested that AICAR treatment resulted in an increase in PK activity and AMPK activity, however, PK activity was lowered by AMPK knockout [20]. These results suggest that AMPK might be involved in the muscle energy metabolism of mice subjected to pre-slaughter stress.

ATP, a high-energy compound in muscle, performs vital cellular functions, such as muscle contraction, cell signaling and biosynthesis of macromolecules [24]. The phosphagen system can be immediately activated in an attempt to maintain ATP stable. During early postmortem metabolism, muscle ATP concentration remains stable through the utilization of a high-energy phosphate compound known as PCr [25]. It has been demonstrated that dietary GAA supplementation could increase the content of PCr in postmortem muscle, which donates its phosphate group to ADP to form ATP [26]. Accordingly, in our present study, an increased ATP content and a decreased AMP content as well as a reduced ratio of AMP to ATP were observed in response to GAA administration when compared with the control group. Similar results were also observed in our previous studies, in which broilers supplemented with GAA exhibited higher ATP content in postmortem muscle [2,15], indicating that GAA could elevate energy store and contribute to delay the rate of ATP depletion. No differences were observed in ATP, ADP and the ratio of AMP to ATP in mice treated with HAT II injection. Similarly, Yan et al [27] demonstrated that the ATP content in the longissimus lumborum muscle of pigs receiving curcumin (an inhibitor of acetyltransferase) treatment was not different from those in the control group. But the AMP/ATP ratio was increased by HAT II injection. Moreover, in comparison with those only receiving GAA administration, AICAR injection increased ATP consumption, evidenced by a higher ratio of AMP to ATP. Additionally, it was well clarified that AMPK can be switched on in response to the increase in AMP/ATP ratio [3], implying that AICAR and HAT II treatment might affect the energy metabolism via the AMPK signaling pathway.

The LKB1 and AMPK proteins participate in an energy sensing cascade that responds to depletion of ATP, and the LKB1/AMPK pathway has been considered as primarily an energy sensing pathway engaged by cells in response to low energy levels [28]. AMPK consists of three subunits (α, β and γ), among which, the α subunit comprises α1 and α2. Moreover, AMPKα2 but not AMPKα1 KO abolished the activity of AMPK in postmortem muscle [1], indicating that phosphorylation of the α2 subunit was more AMP-dependent [29,30]. In accordance with this, our results revealed that GAA treatment downregulated protein abundances of LKB1 and AMPKα2, whereas, AMPKα1 protein level was not affected by GAA administration, suggesting that GAA regulated muscle energy metabolism under stress through the LKB1/AMPK signaling pathway. It has been demonstrated that AMPK, as a cellular energy sensor triggered by falling energy status, can be activated by an increase in AMP/ATP ratio [31,32]. Accordingly, compared with the control group, GAA administration diminished the AMP/ATP ratio and further induced the down-regulation of AMPK signaling in our present study, evidenced by the reduced protein abundance of AMPKα2. The information mentioned above might provide that GAA could inhibit AMPK signaling through the down-regulation of AMP/ATP ratio. In a previous study, it was demonstrated that 50 μg/kg AICAR could activate AMPK signaling pathway and elevate the protein expression of AMPK [7]. Compared with GAA administration, AICAR injection upregulated protein expressions of LKB1 and AMPKα2, but the protein abundance of AMPKα1 was not affected, implying that AMPKα2 could be more sensitive to ATP content than AMPKα1 even under activation by AICAR [33]. Moreover, it was demonstrated that AMPK is a major regulator of postmortem glycolysis and leads to a reduction in the value of pH_24h_ postmortem muscle [1]. In consistent with this, our results found that AICAR injection lowered the value of pH_24h_ in muscle compared with GAA administration group, indicating that the activation of AMPK might accelerate muscle glycolysis and ultimately result in a lower pH_24h_ value. However, the protein expression of LKB1 and AMPKα2 was lower in AICAR+HAT II group that those in the AICAR group, indicating that the activation/inhibition effect of AICAR/HAT II on the regulation of GAA in postmortem muscle glycolysis might be associated with the LKB1/AMPK signaling pathway. Winder et al [34] found that AICAR injection increased AMPK activity in skeletal muscle. Furthermore, it was reported that AICAR treatment increased the activity of AMPKα2 and elevated AMPKα2 phosphorylation level in skeletal muscle [29]. The result of a previous study demonstrated that HAT II treatment could reduce ATP depletion and glycogen breakdown in trapezius, psoas major and semitendinosus muscles before slaughter, and further slowed down LD accumulation and pH decline, which could be due to reduced AMPK activation [7,27]. Indeed, in our present study, HAT II treatment reduced the protein abundance of LKB1 and AMPKα2 compared with those receiving only AICAR injection. Taken together, GAA administration could regulate energy metabolism of postmortem muscle via the AMPK signaling pathway and contribute to alleviating the glycolysis of postmortem muscle.

Accumulated evidence has revealed that protein acetylation was closely associated with the glycolysis of postmortem muscle [35–37] and participated in the regulation of antemortem stress on glycolysis in postmortem muscle [19,38]. Besides, it also has been demonstrated that protein acetylation is related to the regulation of AMPK signaling in the glycolysis of skeletal muscle [7]. In our present study, GAA administration downregulated the protein acetylation level in skeletal muscle of mice. Although direct evidence revealing the regulation of GAA on muscle protein acetylation is not available, it has been demonstrated that the activation of AMPK was involved in the regulation of protein acetylation [39]. Furthermore, there is convincing evidence that phosphorylating AMPK on Thr172 inhibits acetyl-CoA carboxylase and thus elevates acetyl-CoA level (a substrate for HAT enzymes) and the activity of lysine acetyltransferases (known as KATs, and previously termed histone acetyltransferases) [40,41]. Moreover, it has been demonstrated that the activation of AMPK increases the inhibitory phosphorylation of ACC and decreases the conversion of acetyl-CoA to malonyl-CoA, leading to increased protein acetylation [42]. Therefore, we could speculate that GAA administration decreases phosphorylation level of AMPKα2, furtherly reduces the level of acetyl-CoA and downregulates total protein acetylation. Convincing evidence demonstrated that AICAR, the activator of AMPK molecular, increased protein level of AMPK [7,34]. Further study revealed that AICAR treatment elevated the level of protein acetylation [7]. In accordance with this, the increased protein acetylation was induced by AICAR injection in our study compared with the GAA group. However, HAT II administration diminished the increased expression of protein acetylation induced by AICAR. Consistent with this, it was reported that 185 μg/kg HAT II treatment was identified to reduce the protein acetylation level in mice muscle subjected to antemortem-stress [7]. Li et al [19] also suggested that pre-slaughter injection of HAT II lowered the abundance of protein acetylation. Moreover, compared with the control group, the protein acetylation expression in the three groups administered with antemortem GAA oral administration was lower. The information mentioned above indicated that GAA could contribute to alleviating postmortem glycolysis via inhibiting protein acetylation.

## CONCLUSION

Antemortem stress increased energy expenditure, accelerated glycolysis metabolism and ultimately led to a lower ultimate pH in postmortem muscle. However, GAA administration could elevate the level of high energy compounds and reduce the ratio of AMP to ATP, further downregulate the protein abundance of AMPK signaling and protein acetylation involving in energy metabolism of postmortem muscle, and ultimately contribute to attenuating the postmortem glycolysis of antemortem-stress muscle. In conclusion, GAA could contribute to alleviating the glycolysis of antemortem-stress muscle through AMPK signaling and protein acetylation.

## Figures and Tables

**Table 1:** Effects of oral administration with GAA on mice muscle energy status

Items	Control^1)^ group	GAA+AICAR group	GAA group	GAA+AICAR+HAT II group	p-value
ATP	5.34±0.12^b^	5.40±0.13^b^	5.68±0.11^a^	5.53±0.21^ab^	0.029
ADP	1.63±0.16^a^	1.54±0.11^ab^	1.32±0.09^c^	1.40±0.07^bc^	0.009
AMP	0.50±0.02^a^	0.46±0.03^b^	0.38±0.03^c^	0.36±0.02^c^	<0.001
AMP/ATP	0.094±0.004^a^	0.085±0.003^b^	0.066±0.005^c^	0.064±0.003^c^	<0.001

Results are presented as the mean value±standard error of means, and n = 10 for each treatment.

GAA, guandineacetic acid; AICAR, 5-aminoimidazole-4-carboxamide1-β-D-ribofuranoside; HAT II, histoneacetyltransferase II; ATP, adenosine triphosphate; ADP, adenosine diphosphate; AMP, adenosine monophosphate.

1) Control, mice were fed the basal diet; GAA+AICAR, mice were fed the basal diet and treated with GAA oral administration combined with intramuscular injection with AICAR; GAA, mice were fed the basal diet and treated with GAA oral administration; GAA+AICAR+HAT II, mice were fed the basal diet and treated with GAA oral administration combined with intramuscular injec-tion with AICAR and HAT II.

a–c Means in the same row with different superscripts indicate significant difference (p<0.05).
